# Shell colour, temperature, (micro)habitat structure and predator pressure affect the behaviour of *Cepaea nemoralis*

**DOI:** 10.1007/s00114-018-1560-2

**Published:** 2018-05-09

**Authors:** Zuzanna M. Rosin, Zbigniew Kwieciński, Andrzej Lesicki, Piotr Skórka, Jarosław Kobak, Anna Szymańska, Tomasz S. Osiejuk, Tomasz Kałuski, Monika Jaskulska, Piotr Tryjanowski

**Affiliations:** 1 Department of Cell Biology, Faculty of Biology, Adam Mickiewicz University, Umultowska 89, 61-614 Poznań, Poland; 2 Department of Ecology, Swedish University of Agricultural Sciences, Box 7044, Se, 750 07 Uppsala, Sweden; 3 Department of Avian Biology and Ecology, Faculty of Biology, Adam Mickiewicz University, Umultowska 89, 61-614 Poznań, Poland; 4 Institute of Nature Conservation, Polish Academy of Sciences, Mickiewicza 33, 31-120 Krakow, Poland; 5 Department of Invertebrate Zoology, Faculty of Biology and Environmental Protection, Nicolaus Copernicus University, Lwowska 1, 87-100 Toruń, Poland; 6 Department of Behavioural Ecology, Faculty of Biology, Adam Mickiewicz University, Umultowska 89, 61-614 Poznań, Poland; 7 Institute of Plant Protection - National Research Institute, Research Centre of Quarantine, Invasive and Genetically Modified Organisms, Wl. Wegorka 20, 60-318 Poznan, Poland; 8 Department of Entomology, Animal Pests & Biotechnology, Institute of Plant Protection - National Research Institute, Wladyslawa Wegorka 20, 60-318 Poznan, Poland; 9 Institute of Zoology, Poznań University of Life Sciences, Wojska Polskiego 71C, 60-625 Poznań, Poland

**Keywords:** Adaptation, Behaviour, Climatic selection, Climbing, Shelter, Snail

## Abstract

Although shell colour polymorphism of the land snail *Cepaea nemoralis* is a well-known phenomenon, proximate and ultimate factors driving its evolution remain uncertain. Polymorphic species show variation in behavioural responses to selective forces. Therefore, we estimated effects of various environmental factors (temperature, humidity, food availability, (micro)habitat structure and predatory pressure) on behavioural response (frequency of locomotion, climbing and hiding) of *C. nemoralis* morphs, in experimental and natural conditions. In the experimental part of study, the frequency of locomotion was negatively affected by temperature and the presence of food and positively influenced by the presence of light. Morphs significantly differed in behavioural responses to environmental variability. Pink mid-banded and yellow five-banded morphs climbed less often and hide in shelter more often than yellow and pink unbanded individuals when temperature was low and food was absent. Snails fed most often at moderate temperature compared to low and high temperatures. Field investigations partially confirmed differences among morphs in frequency of climbing, but not in terms of probability of hiding in sheltered sites. In natural colonies, temperature and (micro)habitat structure significantly affected frequency of climbing as well as hiding in shelter. Snails more often hid in sheltered sites where thrushes preyed on *Cepaea*. Tendency of unbanded morphs to climb trees may have evolved under avian predatory pressure as thrushes forage on a ground. Tendency of banded morphs to hide in sheltered sites may reflect prey preferences for cryptic background. The results implicate that differential behaviour of *C. nemoralis* morphs compensate for their morphological and physiological limitations of adaptation to habitat.

**Electronic supplementary material:**

The online version of this article (10.1007/s00114-018-1560-2) contains supplementary material, which is available to authorized users.

## Introduction

Knowledge of ecological factors driving genetic spatiotemporal variation in species’ morphological features has been a basis for understanding processes of evolution. For numerous polymorphic species, a clear relation between morph fitness and selective forces has been shown (Endler 1986; Bond 2007). However, in the case of the land snail *Cepaea nemoralis* (L.), a model organism in evolutionary ecology, so much work has been done that the complexity of the problem has been revealed and often no simple answer can be given.

Populations of *C. nemoralis* exhibit genetic variability in shell colour (mainly yellow, pink or brown) and banding pattern (zero to five dark bands sometimes joined together) (Richards and Murray 1975). Many factors have been shown to affect *Cepaea* morph fitness including selective predation and (micro)climatic selection (e.g. Cain and Sheppard 1950; Cook 1998, 2005; Cameron and Pokryszko 2008; Silvertown et al. 2011; Ożgo 2012; Cook 2013; Schilthuizen 2013). Furthermore, landscape structure and history have been used to explain morph frequency variation in terms of heterozygote advantage or heterosis and drift (Cain and Sheppard 1954; Cook 1998; Cook 2007; Le Mitouard et al. 2010; Pokryszko et al. 2012). Selective predation and climatic selection seem the most popular hypotheses, though (Cain and Sheppard 1961; Cook 1998, 2013). The former is based on the variation in conspicuousness and/or shell strength of *Cepaea* morphs (Cain and Sheppard 1954; Cook 1986; Rosin et al. 2013) and frequency-dependent selection by predators (Clarke 1969; Allen and Weale 2005; Holmes et al. 2017). The latter hypothesis assumes variation in morph survival in different habitats and at different latitudes because of the variation in thermal properties of morph shells (Heath 1975; Jones et al. 1977; Tilling 1983; Ożgo 2012).

As Livshits (1978, 1981) has shown, morphs in polymorphic species may vary in their behavioural response to potentially selective environmental factors. The behaviour of *Cepaea* snails is strongly dependent on environmental cues of light, temperature and humidity (Wolda 1965; Cameron 1970a, b; Jaremovic and Rollo 1979; Lima and Dill 1989; Ożgo and Kubea 2005). Climbing, hiding and microhabitat selection may all operate to protect the snails from predation (Lefcort et al. 2006). As the thermal properties and conspicuousness of the colour and banding morphs differ, we might expect that there would also be different behavioural adjustments among morphs to any given set of environmental conditions. While Chang and Emlen (1993) and Ożgo and Kubea (2005) provide some evidence that this is indeed the case, and Tilling (1983) has shown experimentally that thermal properties affect behaviour and mortality, there is a dearth of corroborative evidence on this matter. The evidence for selective differences among morphs with respect to climate or microclimate comes mainly from correlative studies across many populations (e.g. Jones 1973; Jones et al. 1977; Ożgo 2005). The variable behaviour of different morphs might therefore contribute to the maintenance of polymorphism in heterogeneous environments, such as those often occupied by *Cepaea* snails (Cook 1998). Thus, we expected that differences in morphology of *Cepaea* morphs should be reflected in behavioural variation increasing snails’ adaptation to a range of environmental conditions including predatory pressure (Wcislo 1989).

The aim of this study has been to test differences in behaviour of *C. nemoralis* morphs (frequency of inactivity, locomotion, climbing up tall objects, hiding in shaded sites) in relation to environmental variability (temperature, food availability, (micro)habitat structure and predation pressure), both in experimental and natural conditions. We expected that yellow and unbanded morphs would be more active at higher temperatures and climb more often in comparison to pink and banded (i.e. darker) snails (Wolda 1965; Ożgo and Kubea 2005). We also predicted that various environmental factors would affect the behaviour of *Cepaea* morphs differently, according to the assumption that morphs’ fitness varies with changing environmental conditions (Livshits 1978, 1981).

## Material and methods

### Laboratory experiments

#### Material collection

Snails were collected in September 2011 from a population located near the city of Poznań, Wielkopolska, Poland. The sampling site covered 400 m^2^, which is the estimated size of one panmictic unit in *C. nemoralis* (Lamotte 1951). Specimens were collected from a diversified site where vegetation has been spontaneously developing for over 50 years. Our study area was dominated by open habitats composed mainly of grasses (about 40% of the site area) and psammophilic vegetation with dwarf everlast (*Helichrysum arenarium* (L.); about 30% of the site area). Dark, shaded habitats (ca. 30% of the area) were composed of shrubs, including blackthorn (*Prunus spinosa* L.), and trees: black locust (*Robinia pseudoacacia* L.) and hedge maple (*Acer campestre* L.). The collection of snails was random. Altogether, 40 snails were collected with ten specimens of the following morphs: pink unbanded, pink mid-banded, yellow unbanded and yellow five-banded. Brown-shelled snails were not present. All specimens were adult and in a good condition (intense shell colour, lack of shell damages, fully developed labium).

#### Study procedure

Collected snails were kept in plastic, aerated boxes at 22 °C and humidity of 80–90% without food (to empty their alimentary canals) for 24 h after the capture. Next, the snails were weighed with a scale (to the nearest 0.01 g) and individually marked. Immediately before the start of the experiment, snails were placed in aerated boxes (dimensions 52 × 37 × 33 cm). Boxes were made of semitransparent plastic and their covers equipped with infrared video cameras (CCTV 720 × 480 pixels). Snail behaviour was recorded constantly, at a constant frame rate of 12.5/s.

**Fig. 1:**
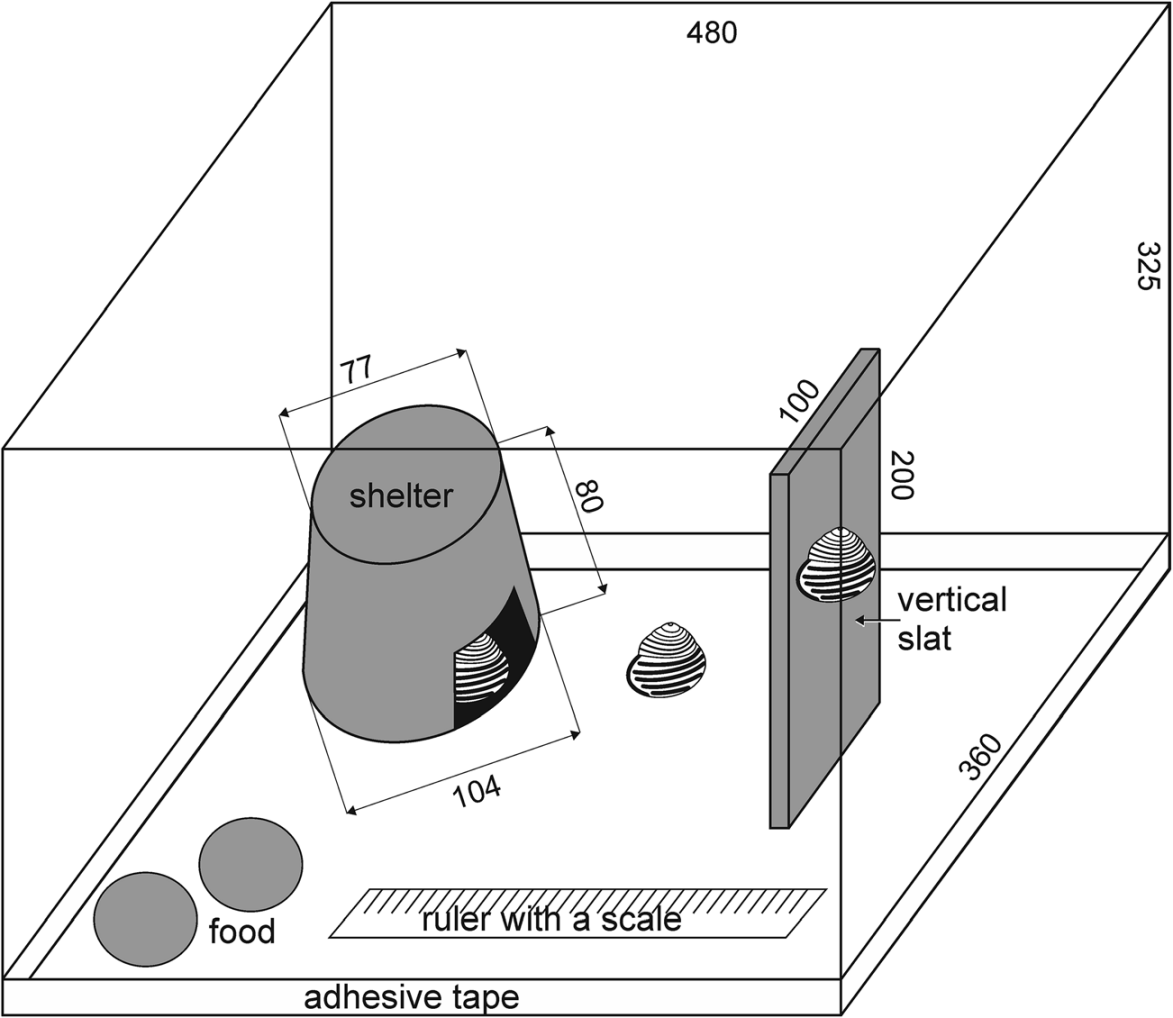
Illustration of an experimental box used in laboratory part of the study. Dimensions are presented in milimeters

Due to questionable identification of morph colour in conditions of dim light, each morph (10 specimens per morph) was placed in a separate, randomly assigned box. The bottom of boxes was covered with 5-cm-thick layer of soil. The snails were prevented from climbing up the walls of boxes by adhesive tape plastered along the walls and covered with salt. The boxes were equipped with a small plastic box (hereafter: “shelter”) placed on the bottom to provide the snails with a shaded shelter and a transparent plastic slat up which the snails could climb (Fig. 1). The external surface of the shelter also enabled climbing. The experimental boxes with snails were located in a laboratory incubator equipped with a thermostat and photoperiod regulator (12-h light: 12-h darkness during experiment). Humidity was controlled and maintained at constant level (90 ± 3%).

Temperature (7, 17 and 27 °C), light (absence [0] or presence [1]) and food ([0] or [1]) varied during the experiment. Two replicates of experimental conditions were made. In each replicate, 48 h covered each temperature category and this period was divided into 24 h with food available and 24 h without food (cucumber and cabbage slices, about 5 or 7–8 cm in diameter, respectively). Each 24-h period consisted of a 12:12-h light:dark cycle. Therefore, one replicate lasted for 144 h for each morph. Replicates started at 10:00 on the first day and ended at 10:00 on the seventh day. The minimum interval between replicates was 2 days. The snails were sprayed with water (to bring them back to full activity) once a day at 10:00 and placed in the box corner; ground was also moistened (with equal amount of water). The sequence of temperature categories was random in each replicate. Morphs were randomly assigned to boxes in each replicate. Dependent variables were the following behaviour types:

- Activity, with four categories: 0—being inactive (entire body withdrawn into the shell), 1—foot everted, 2—foot and head everted, 3—locomotion
- Position in the box with three categories: (i) ground surface, (ii) inside the shelter box and (iii) above the ground (the plastic slat and the external surface of small box)
- Feeding—two categories: present or not

Different activities might have been performed in different locations; for instance, snails might move on the ground or tall objects—then were scored as “locomotion” in term of activity categories, and “on the ground” or “above the ground”, respectively, in terms of position in the box. In the case of snails staying in a shelter, activity was not scored as following them was impossible. Snails fed always on the ground, with head and foot everted.

#### Video analysis

Altogether, 1,152 h of recordings were analysed (2 × 144 h per morph). Behaviour was scored in 15-min intervals (720 records per morph and replicate; no breaks between intervals) in the panel of Observer Pro 5.0 (Noldus 2003). Scores for snail behaviour (10 snails per morph) recorded in intervals were then averaged over 4-h periods. The data were expressed as mean number of occurrences of a given behaviour per minute per ten individuals in a 4-h period (excluding time spent in the shelter from the calculation of activity-related variables, i.e. inactivity and locomotion) (Table S1). The order of subsequent periods in each replicate was noted and taken to further analyses as an explanation of change in snail behaviour with time. Due to some difficulties in recognising individual snail identity, we decided to determine the total number of snails in the box showing a given behaviour (according to the types and categories described above).

### Field observations

#### Research areas

Surveys were conducted in two large plots located in rural landscapes in the vicinities of Gdańsk (54° 20′ N, 18° 38′ E; plot area 251 km^2^) and Poznań (52° 26’ N, 16° 52′ E; 252 km^2^) (Rosin et al. 2017). *Cepaea nemoralis* colonies were searched for in randomly selected potential habitats (fallow lands, hedges, ecotonal fragments of forests). Altogether, 56 colonies were studied. As the annual dispersion distance of *C. nemoralis* is 10 m (Lamotte 1951), we regarded two colonies as separate if the distance between them was at least 100 m. Maximum distance between colonies within the studied landscapes was 15,800 m. Colony area subjected to surveys ranged from 100 to 500 m^2^. Majority of colonies inhabited wastelands (86% of colonies) with variable density of trees and bushes (0–50%) and only few—ecotones, forests and orchards (14%).

#### Data collection

Each colony was surveyed three times: between 1st May and 30th June, 1st July and 31st August, 1st September and 31st October 2012 (Rosin et al. 2017). The minimal interval between visits in a colony was 30 days. Surveys were conducted in conditions that were favourable for snail activity (between 06:00 and 11:00). In each colony, the snails were counted in 50 cm × 50 cm squares, spaced 5 m apart along a transect of 20 m (5 squares per transect). Depending on the colony area, the number of transects ranged from 1 to 5, and the distance between adjacent transects was 5 m. The location of the square plots and the number of transects in a colony varied across visits, and the latter also depended on colony area (Rosin et al. 2017). Each live specimen was scored for shell colour (yellow, pink or brown) and banding pattern (zero, one, three or five). Climbing and hiding in shade behaviours were noted. Climbing determined whether a snail was located on an above-ground object or on the ground level. The height of its position was also noted (including climbing on trees, which was observed with binoculars). Hiding in shade was determined on the basis of 50 × 50 cm plot where a snail was found—whether it was located in shade or not. Two main groups of explanatory variables corresponding to (1) predation and (2) (micro)habitat structure were determined.

#### Predation

Rodent holes were counted in 50 × 50 cm squares as an indicator of rodent abundance (Mitchell and Balogh 2007). Pairs of thrushes (song thrush *Turdus philomelos*, blackbird *T. merula*, fieldfare *T. pilaris*, mistle thrush *T. viscivorus*, being main predators of *C. nemoralis*) breeding within a 50-m radius from a colony were also counted (Table S2; Rosin et al. 2017).

Moreover, shells showing traces of predation by birds or rodents were counted in squares as direct evidence of predation. Shell damage was attributed to birds based on marks of strikes on the left part of the last whorl, whereas rodent marks were identified based on teeth marks near the aperture (Rosin et al. 2011). Predation was estimated during each visit in a snail colony.

#### (Micro)habitat type and structure

Microhabitat structure was estimated within 50 × 50 cm squares in which the snails were surveyed (Rosin et al. 2017). Each square was determined as shaded (if were located under tree or shrub) or open (outside the area shaded by tree or shrub). The mean height of vegetation (based on five random measurements) and the percentage cover of dead and alive vegetation as well as of bare ground were also determined in each square (Table S2). During each survey, temperature [°C] and humidity [%] were determined using a portable weather station (La Crosse WS2355). Moreover, we noted the distance of each square to the nearest shrub and tree (Table S2; Rosin et al. 2017). Most correlations between environmental variables were weak (Table S3).

### Statistical analyses

#### Laboratory experiments

Dependent variables subjected to statistical analyses included frequencies of (1) inactivity, (2) locomotion, (3) staying on the ground, (4) staying in the shelter box, (5) climbing and (6) feeding. Remaining activity categories (foot everted or foot and head everted) were observed at marginal frequencies. Dependent variables were strongly correlated to each other; thus, a principal component analysis (PCA) was applied to get a set of new uncorrelated variables. Three axes explained altogether 87.6% of the observed variation (Table 1). The first principal component (PC1) was negatively related to frequency of locomotion, staying on the ground and feeding and depended positively on frequency of being inactive; thus, it was indication of inactivity. The second principal component (PC2) was strongly negatively correlated with frequency of climbing and positively related to frequency of staying in shelter. In turn, PC3 was negatively correlated with frequency of feeding (Table 1). These three principal components described snail behaviour and were used as dependent variables in linear mixed models (LMEs) with the effects of morph, temperature, light, food and order of consecutive 4-h measurements (a continuous variable accounting for the impact of passing time on snail behaviour) as well as their interactions up to the third order (Table 2). Replicate identity was assigned as a random factor. As the same snails were used in consecutive treatments, there was a problem with data dependence. Namely, behaviour of individuals in samples separated by small time span was perhaps more similar than that in timely more distant samples. To account for this, we used a linear mixed model with a temporal autocovariate (Turchin 1998). Linear mixed models were built separately for each principal component. The function *lme*() in “nlme” package (Pinheiro et al. 2017) implemented in R (R Core Team 2017) was used for this purpose.

**Table 1:** Principal component analysis of variables describing behaviour of *Cepaea nemoralis* in laboratory conditions. Correlations between variables and principal components higher than 0.4 are italicised. Abbreviations of behaviour types: Inactive—frequency of being inactive, Locomotion—frequency of locomotion, On the ground—frequency of staying on the ground, Above the ground—frequency of staying above the ground (on tall objects), Shelter—frequency of staying in the shelter, Feeding—frequency of feeding

Variable	PC1	PC2	PC3
Inactive	*0.569*	0.1	− 0.035
Locomotion	*− 0.526*	− 0.105	0.287
On the ground	*− 0.471*	− 0.012	0.215
Above the ground	0.295	*− 0.649*	0.002
Shelter	0.030	*0.727*	− 0.165
Feeding	− 0.300	− 0.171	*− 0.918*
Variance explained (%)	44.9	28.9	13.9

**Table 2:** Linear mixed model (with temporal autocorrelation) of the effects of morph, temperature, presence/absence of light and food, time (order) and their interactions on behaviour of *C. nemoralis* described by principal components (PC1, PC2 and PC3, see Table 1). Degrees of freedom for the effect (df) and error (df den) are shown. Significant effects are italicised

PC	Predictor	df	df den	*F*	*P*
1	Morph (M)	3	260	2.081	0.103
	*Temperature (T)*	2	260	14.704	*< 0.001*
	*Light (L)*	1	260	28.720	*< 0.001*
	Food (F)	1	260	2.166	0.142
	*Order (O)*	1	260	4.817	*0.029*
	M × T	6	260	0.699	0.651
	M × L	3	260	0.842	0.472
	M × F	3	260	0.618	0.604
	T × L	2	260	1.194	0.305
	T × F	2	260	1.840	0.161
	L × F	1	260	0.109	0.742
	M × O	3	260	0.379	0.768
	T × L × M	6	260	0.288	0.943
	T × F × M	6	260	0.975	0.442
	L × F × M	3	260	0.329	0.804
2	*Morph (M)*	3	260	4.579	*0.004*
	*Temperature (T)*	2	260	11.425	*< 0.001*
	Light (L)	1	260	0.405	0.525
	*Food (F)*	1	260	6.473	*0.012*
	*Order (O)*	1	260	25.523	*< 0.001*
	M × T	6	260	1.504	0.177
	M × L	3	260	0.033	0.992
	*M × F*	3	260	3.175	*< 0.025*
	*T × L*	2	260	3.651	*< 0.027*
	*T × F*	2	260	8.469	*< 0.001*
	L × F	1	260	1.069	0.302
	M × O	3	260	1.418	0.238
	T × L × M	6	260	1.029	0.407
	*T × F × M*	6	260	4.983	*< 0.001*
	L × F × M	3	260	0.413	0.744
3	Morph (M)	3	260	0.311	0.817
	*Temperature (T)*	2	260	4.462	*0.012*
	Light (L)	1	260	0.071	0.789
	*Food (F)*	1	260	23.706	*< 0.001*
	Order (O)	1	260	2.674	0.103
	*M × T*	6	260	2.320	*0.034*
	M × L	3	260	0.780	0.506
	M × F	3	260	1.778	0.152
	T × L	2	260	0.407	0.666
	*T × F*	2	260	7.900	*< 0.001*
	L × F	1	260	2.649	0.105
	M × O	3	260	0.919	0.432
	T × L × M	6	260	1.094	0.366
	T × F × M	6	260	1.368	0.228
	L × F × M	3	260	2.557	0.056

#### Field data

Percentage of snails being at least 5 cm above the ground was low (12.3% of all individuals) and thus insufficient to test the effects of morph and environmental variables on climbing height using linear models. Instead, single-variable and multifactor generalised linear mixed models (GLMMs) with binomial distribution and logit link function were applied to relate environmental variables to frequency of climbing behaviour*.* We used morph type, temperature, humidity, covers of alive plants, dead vegetation and bare ground, vegetation height, presence/absence of shade, distances to the nearest shrub and tree, presence/absence of shells damaged by birds and rodents as well as density of birds (pairs of thrushes) and rodent holes as explanatory fixed effects. First, we built a set of GLMMs with single variables. Secondly, we built a multifactor model with those variables which were statistically significant in the single-variable models. Landscape (Poznań, Gdańsk), survey, colony (nested in a landscape) and square identity (nested in a colony) were assigned as random effects. We also calculated marginal *R*^2^ and conditional *R*^2^ in all GLMMs according to Nakagawa and Schielzeth (2013). The probability of hiding in shade was analysed in an analogical manner. These analyses were performed in “lme4” statistical package (Bates et al. 2015) in R.

## Results

### Effects of morph type, temperature, light, food and time on *C. nemoralis* behaviour in experimental conditions

**Fig. 2:**
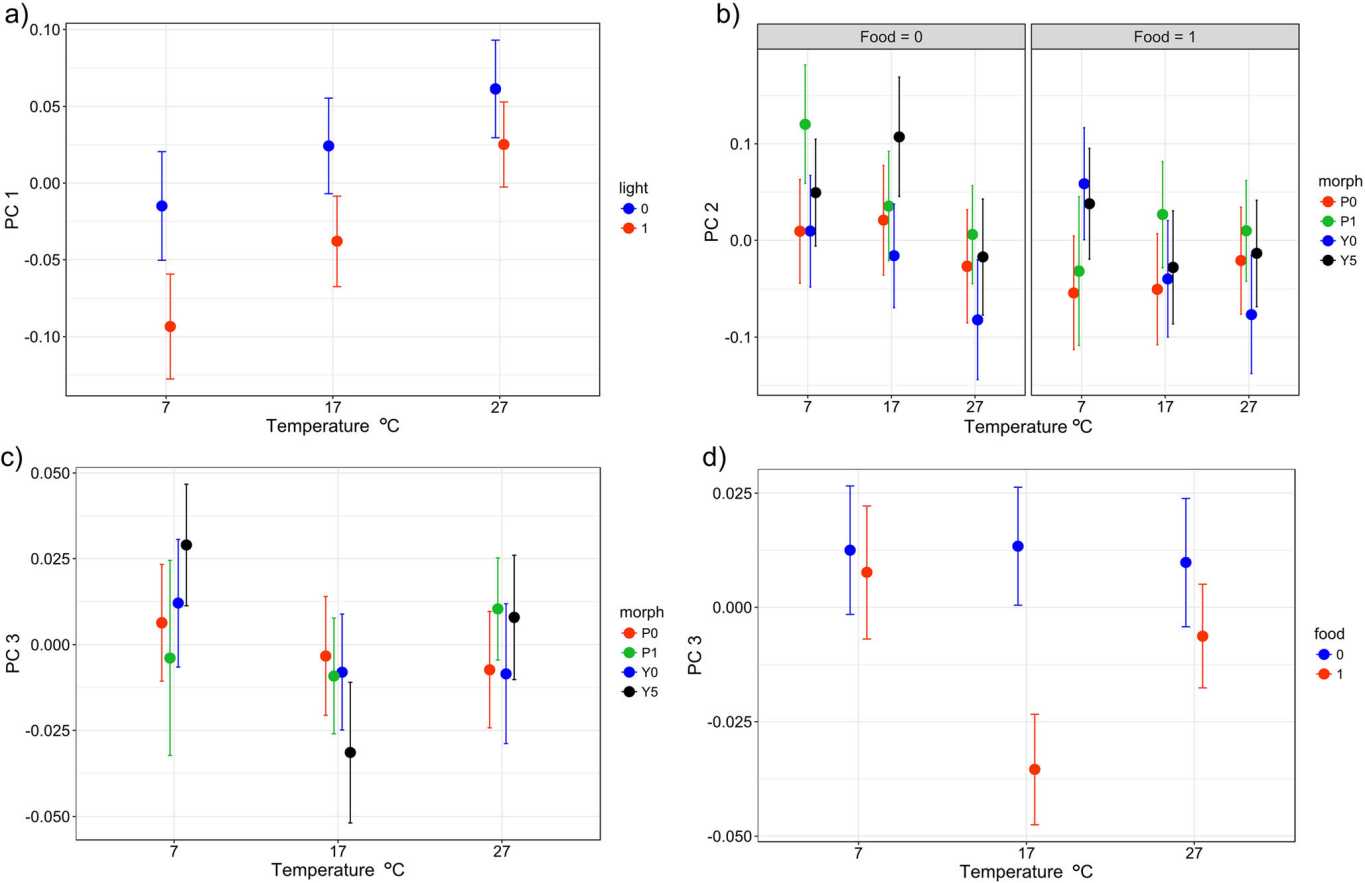
Relationships between various behaviour types of *C. nemoralis* described by PC1, PC2 and PC3 and explanatory variables studied in laboratory conditions. **a** Mean PC1 (positively correlated with frequency of being inactive and negatively associated with frequency of locomotion and staying on the ground) in relation to temperature and light conditions. **b** Mean PC2 (negatively correlated with frequency of climbing and positively associated with frequency of hiding in shade) in relation to temperature × food × morph interaction. **c** Mean PC3 (negatively correlated with frequency of feeding) in relation to temperature × morph interaction. **d** PC3 in relation to food × temperature interaction. Means (solid circles) ± 95% confidence intervals (whiskers) estimated from linear mixed models are presented

The most common behaviours of snails were inactivity and staying above the ground (Table S1). The values of PC1 (based on frequency of being inactive and staying on the ground) were significantly positively influenced by temperature and order (time) and negatively affected by light presence (Table 2, Fig. 2a). Variation of principal component 2 (frequency of staying above the ground and in the shelter) was significantly affected by an interaction temperature × food × morph (Table 2). Pink mid-banded and yellow five-banded morphs hid in the shelter more often (and stayed above the ground less often) than yellow and pink unbanded snails when temperature was low and food was absent (Fig. 2b). PC3 (frequency of feeding) was significantly affected by morph × temperature and temperature × food (Table 2, Fig. 2). The first interaction indicated that at low temperatures, yellow five-banded morphs fed more often than other morphs but the opposite was found at moderate temperatures (Fig. 2c). The interaction between temperature and food indicated that values of PC3 were similar when food was absent and present at low and high temperatures but PC3 was lower when food was present at moderate temperatures (Fig. 2d).

Order of sampling periods was significant in model for PC1 and PC2 (Table 2). Snails moved less often and stayed in shelter more often with time of the experiment.

### *C. nemoralis* behaviour in natural conditions

We found 3,254 snails with the most frequent yellow mid-banded and five-banded as well as pink mid-banded morphs (Table S4). Generally, yellow unbanded snails climbed up and stayed in shade more often than the remaining morphs (Table S4).

#### Climbing behaviour

Snails climbed up on average 44.03 cm (± SD = 46.76 cm) above the ground with the height range varying from 5 to 250 cm. The GLMM showed that the probability of snail climbing behaviour was dependent on morph type, as well as on height of vascular vegetation, presence of shade and temperature (positive relationships) and distance to the nearest shrubs (negative relationship) (Table 3). Yellow unbanded, pink mid-banded and brown mid-banded morphs climbed more frequently than yellow five-banded ones (Tables 4 and S4).

**Table 3:** Single-variable and multifactor generalised linear mixed models (GLMMs) with binomial distribution and logit link function on predictors of occurrence *Cepaea nemoralis* climbing behaviour in 50 × 50 cm plots within its colonies*.* Test statistics (*F*) with degrees of freedom (df) and significances (*P*) for single-factor analyses and multifactor models with marginal and conditional *R*-squared variance (*R*^2^_m_, *R*^2^_c_, respectively) explained by models. Statistically significant effects are italicised. Explanation of variable codes: Morph—a morph type; Dry plant—covers of dead, dry vegetation; Alive plant—cover of alive plants; Bare ground—cover of bare/ground; VegHeight—mean vegetation height; Shade—describes shading (%) of the plot; DistShrub—distance to nearest shrub; DistTree—distance to nearest tree; Thrush number—number of breeding pairs of thrushes (*Turdus* sp.); DamBird—presence of snail shells damaged by birds; DamRodent—presence of snail shells damaged by rodents; Rodent holes—the number of rodent holes

Variable	Single-variable GLMMs					Multifactor GLMM (*R*^2^_m_ = 0.29, *R*^2^_c_ = 0.48)			
	*R*^2^_m_, *R*^2^_c_	Slope (SE)	*F*	df	*P*	Slope (SE)	*F*	df	*P*
Morph	0.12, 0.18	–	2.708	9, 324	*0.004*	–	*2.559*	*9, 3237*	*0.006*
Dry plant	0.00, 0.17	− 0.002 (0.004)	0.196	1, 185	0.658				
Alive plant	0.00, 0.16	0.006 (0.004)	1.816	1, 141	0.178				
Bare ground	0.02, 0.17	− 0.015 (0.005)	7.430	1, 325	*0.006*	− 0.009 (0.006)	2.536	1, 217	0.111
VegHeight	0.02, 0.21	0.014 (0.004)	10.058	1, 125	*0.002*	0.014 (0.005)	9.892	1, 913	*0.002*
Shade	0.09, 0.21	− 1.316 (0.255)	26.610	1, 812	*< 0.001*	− 1.155 (0.261)	19.590	1, 757	*< 0.001*
DistShrub	0.16, 0.27	− 0.033 (0.012)	7.348	1, 492	*0.007*	− 0.029 (0.013)	4.852	1, 531	*0.028*
DistTree	0.17, 0.30	− 0.039 (0.012)	11.256	1, 345	*0.001*	− 0.013 (0.03)	12.921	1, 324	< 0.001
Thrush number	0.01, 0.17	− 0.375 (0.135)	7.752	1, 128	*0.005*	0.047 (0.140)	1.111	1, 319	0.737
DamBird	0.00, 0.17	− 0.059 (0.502)	0.014	1, 947	0.907				
DamRodent	0.01, 0.19	0.138 (0.277)	0.124	2, 325	0.554				
Rodent holes	0.00, 0.17	− 0.194 (0.212)	0.863	1, 143	0.361				
Temperature	0.15, 0.22	0.134 (0.019)	51.788	1, 323	*< 0.001*	0.128 (0.020)	39.836	1, 222	*< 0.001*
Humidity	0.00, 0.06	− 0.004 (0.009)	0.214	1, 738	0.644

**Table 4:** Estimates of the function slopes for the effect of morph in GLMM on snail climbing, presented in Table 3. The yellow 5-banded morph was set as a reference category. Statistically significant differences between yellow five-banded and listed morphs are italicised

Morph	Estimate	SE	*t*	*P*
Brown unbanded	0.482	0.280	1.723	0.085
Brown mid-banded	1.316	0.639	2.059	*0.040*
Pink unbanded	0.315	0.244	1.295	0.195
Pink mid-banded	0.646	0.196	3.296	*0.001*
Pink three-banded	− 0.167	0.363	− 0.460	0.645
Pink five-banded	− 0.187	0.338	− 0.554	0.580
Yellow unbanded	0.627	0.204	3.072	*0.002*
Yellow mid-banded	0.250	0.191	1.308	0.191
Yellow three-banded	− 0.594	0.415	− 1.432	0.152
Yellow five-banded	0			

#### Hiding in shade

The GLMM analysing probability of staying in shade showed that morph had no statistically significant effect on this behaviour. Probability of staying in shade was positively correlated with the presence of shells damaged by birds (Table 5). Moreover, this variable was positively correlated with cover of dry plants, vegetation height, temperature and, of course, shading of plots (Table 5). Also, when trees or shrubs were distant from plots, then probability of staying in shade decreased (Table 5).

**Table 5:** Single-variable and multifactor generalised linear mixed models (GLMMs) with binomial distribution and logit link function on predictors of *Cepaea nemoralis* hiding in shade in 50 × 50 cm plots within its colonies*.* Test statistics (*F*) with degrees of freedom (df) and significances (*P*) for single-factor analyses and multifactor models with marginal and conditional *R*-squared variance explained by models. Statistically significant effects are in italics. Explanation of variable codes: see Table 3

Variable	Single-variable GLMMs					Multifactor GLMM (*R*^2^_m_ = 0.58, *R*^2^_c_ = 0.84)			
	*R*^2^_m_, *R*^2^_c_	Slope (SE)	*F*	df	*P*	Slope (SE)	*F*	df	*P*
Morph		–	1.724	9, 322	0.078	–	1.308		0.227
Dry plant	0.01, 0.76	0.017 (0.003)	29.474	1, 325	*< 0.001*	0.011 (0.005)	5.284	1, 3236	*0.022*
Alive plant	0.02, 0.75	− 0.016 (0.003)	29.617	1, 325	*< 0.001*	− 0.008 (0.005)	2.590		0.108
Bare ground	0.00, 0.01	0.002 (0.004)	0.232	1, 325	0.630				
VegHeight	0.02, 0.75	0.006 (0.002)	6.462	1, 325	*0.011*	0.012 (0.003)	16.763	1, 3252	*< 0.001*
Shade	0.22, 0.63	0.062 (0.007)	82.329	1, 291	*< 0.001*	0.055 (0.008)	53.146	1, 291	*< 0.001*
DistShrub	0.03, 0.74	− 0.039 (0.009)	17.729	1, 325	*< 0.001*	− 0.051 (0.012)	19.497	1, 3252	*< 0.001*
DistTree	0.37, 0.81	− 0.168 (0.017)	103.118	1, 317	*< 0.001*	− 0.134 (0.016)	70.630	1, 3252	*< 0.001*
Thrush number	0.01, 0.75	− 0.173 (0.115)	2.266	1, 325	0.132				
DamBird	0.04, 0.73	1.562 (0.341)	20.919	1, 325	*< 0.001*	2.026 (0.417)	23.619	1, 3252	*< 0.001*
DamRodent	0.00, 0.7	0.117 (0.242)	0.233	1, 325	0.630				
Rodent holes	0.00, 0.75	0.250 (0.166)	2.272	1, 325	0.132				
Temperature	0.01, 0.75	0.069 (0.014)	23.026	1, 780	*< 0.001*	0.033 (0.017)	3.969	1, 78	*0.046*
Humidity	0.00, 0.75	0.001 (0.006)	0.050	1, 325	0.823				

## Discussion

Our study revealed that *C. nemoralis* exhibited a complex behaviour in response to a set of environmental factors. We found differences between morphs and significant effects of various environmental factors on *Cepaea* behaviour. We also showed that snail behaviour was significantly affected by interactions between morph type and environmental variables. These indicate that (micro)habitat structure shapes morph behavioural responses to environmental factors.

### Climbing up tall objects

Our results have not confirmed previous findings that yellow morphs climb up more often than the other ones (Wolda 1965). We have shown that unbanded and mid-banded forms climbed up more often than those with three or five bands. Classic studies of Cain and Sheppard (1950, 1954) showed that birds prey upon most frequently on the yellow unbanded morph (especially in spring). Moreover, banded morphs of *C. nemoralis* have thicker shells than unbanded ones (Rosin et al. 2013), which can better protect a snail against predation (e.g. Lewis and Magnuson 1999; Smallegange and Van Der Meer 2003). Therefore, the varying tendency for climbing among morphs may reflect differences in their resistance to predators.

Climbing up is a common behaviour in terrestrial gastropods (e.g. Wolda 1965; Potts 1975; Jaremovic and Rollo 1979). Undoubtedly, this behaviour is energetically very costly and additionally exposes a snail to water loss connected to mucus production necessary for locomotion (Jaremovic and Rollo 1979). Climbing up serves several functions, such as predation avoidance (Lefcort et al. 2006), protection against overheating on bare ground (Pomeroy 1968; Jaremovic and Rollo 1979) and scavenging algae growing on tree bark (Schilthuizen M., personal observation). In the field conditions, snails significantly more often climbed up with increasing temperature, which confirms the role of this behaviour in thermoregulation. We did not find strong evidence for relation of this behaviour with predation. However, this result should be tested in another experimental design, as we used only potential predatory pressure in the field.

### Hiding in shelter

It was shown that brown and banded morphs absorb significantly more thermal energy than yellow and unbanded ones, achieving higher body temperature when exposed to direct solar radiation (Heath 1975; Chang 1991). Consequently, in conditions of high temperature, dark colouration may be physiologically disadvantageous (Heath 1975; Chang 1991). Some studies suggest a behavioural variation among morphs in response to temperatures within colonies, which leads to morph-specific habitat preferences: the yellow morph preferring open, sunny sites, and darker pink and brown forms preferring neighbouring woodland habitats (Cain and Sheppard 1954; Jones 1974; Ożgo 2005; Cook 2008; Cameron and Cook 2012). At the regional scale, morph frequencies in *C. nemoralis* show a latitudinal gradient, with the frequency of yellow morphs increasing southwards in Europe (Jones et al. 1977). Moreover, changes in morph frequencies during the last decades were suspected to be caused by global warming, which was, however, confirmed only partially (Silvertown et al. 2011; Cameron and Cook 2012; Ożgo and Schilthuizen 2012). However, the latest studies on shell thermal capacity conducted in standardised conditions on a polymorphic snail *Theba pisana* showed contrasting results for differences between pale and dark banded morphs (Scheil et al. 2012; Knigge et al. 2017). Nevertheless, this relationship may be more subtle depending on such microhabitat features as vegetation height or alive plant cover (Wolda et al. 1971; Chang and Emlen 1993; Moreno-Rueda 2007).

In our experimental conditions, at low temperature and with food absent, pink mid-banded and yellow five-banded morphs tended to stay in the shelter more often and climbed less often than yellow and pink unbanded individuals. Otherwise, in the field, snails were present in shelters significantly more often with increasing temperature and there was no significant effect of morph type. Our laboratory results suggest that other factors may also influence morph distribution in natural colonies, e.g. food distribution (Wolda et al. 1971; Chang and Emlen 1993). However, our data from experimental part of the study had a degree of non-independence between sample units, which, however, has been at least partially accounted for in statistical modelling and should not affect behavioural differences found between morphs. Theoretically, the preference of banded snails for shaded sites may be the consequence of prey preference for cryptic background (Endler 1981) and/or sites sheltered from sunlight (Chang 1991). Indeed, banded morphs of *Cepaea* are more conspicuous for avian predators than unbanded forms (Surmacki et al. 2013). They are also more susceptible to weight loss and energy metabolism incompatibility when exposed to sunlight and high temperatures, respectively (Steigen 1979; Chang 1991). Moreover, we found that after controlling for other environmental variables, snails more often were present in shelter when shells damaged by birds were present in field study plots. Therefore, hiding in shade may be some kind of response of *Cepaea* to predator activity. However, it also can be a result of selection that favoured snails living in shelters. Thrushes, being main predators of *Cepaea*, use mostly visual clues during foraging; thus, prey staying in shaded sites may be difficult to detect. Alternatively, the found patterns in hiding and climbing behaviour can be due to learning during a specimen’s lifespan and not necessarily have a genetic basis (Loy et al. 2017). The learning may be related to chemical clues left by other individuals that can be followed by others (O’Connell 1986).

The probability of hiding in shelter was positively related to the percentage cover of dry plants in study field squares, indicating that dry dead vegetation may be a stressful substrate for snails because of the lower humidity. Mean vegetation height was also positively related to the tendency of staying in shade of shrubs and trees, contradicting our expectations that high herbaceous plants may serve as a shelter alternative to shrubs and trees in open sites (Chang and Emlen 1993).

### Effects of environmental factors on *C. nemoralis* activity: locomotion and feeding

Snails moved less often with increasing temperature, probably avoiding overheating and excessive water loss (Cameron 1970a, b; Herreid and Rokitka 1976). The presence of light positively influenced frequency of locomotion. In natural conditions, high activity of snails at night compared to diurnal conditions may be driven by relatively low temperature and high humidity at night (Cameron 1970a, b). Studied morphs did not differ significantly in frequency of inactivity and locomotion. It was shown, however, that in conditions of low humidity and direct solar radiation, yellow morphs are active longer than brown ones (Ożgo and Kubea 2005) and unbanded forms are active longer than five-banded ones (Chang 1991). Snails fed most frequently at the moderate temperature (17 °C), which indicates that such conditions are the most favourable for them. This pattern was most marked in yellow five-banded morph, likely due to its high susceptibility to higher temperatures (Chang 1991).

## Concluding remarks

Observed differences in behaviour of *C. nemoralis* morphs may be the result of adaptations compensating morphological and/or physiological limitations affecting morph fitness in a given habitat. These differences may have evolved due to varying pressure of climatic factors and predation pressure on various *Cepaea* morphs. However, the effect of (social) learning during a lifespan of a specimen is also possible. Tendency of unbanded morphs to climb up trees may have evolved under avian predatory pressure. On the other hand, tendency of banded forms to hide in sheltered sites may reflect prey preferences for cryptic background as well as for microhabitats less exposed to sunshine. Our results indicate that relationships between morph frequency and habitat type and/or predation may depend not only on animal colour but also on its behaviour and resource availability. Observed differences in morph behaviour may sustain the stable polymorphism in *C. nemoralis* colonies (Levene 1953; Chang and Emlen 1993).

## Electronic supplementary material

ESM 1 (DOCX 23 kb)
