# Supplementary material for: Shell colour, temperature, (micro)habitat structure and predator pressure affect the behaviour of Cepaea nemoralis
Source: Naturwissenschaften. 2018 May 9;105(5):35. doi: 10.1007/s00114-018-1560-2 (PMC5942350; doi:10.1007/s00114-018-1560-2)
Supplement: Supplementary file 1 — (DOCX 23 kb) [file 114_2018_1560_MOESM1_ESM.docx]

**Electronic Supplementary Material**

**Shell colour, temperature, (micro)habitat structure and predator pressure affect behaviour of *Cepaea nemoralis***

**The Science of Nature**

Zuzanna M. Rosin^1,2^, Zbigniew Kwieciński^3^, Andrzej Lesicki^1^, Piotr Skórka^4^, Jarosław Kobak^5^ Anna Szymańska^6^, Tomasz S. Osiejuk^6^, Tomasz Kałuski^6^, Monika Jaskulska^7^, Piotr Tryjanowski^8^

^1^ Department of Cell Biology, Faculty of Biology, Adam Mickiewicz University, Umultowska 89, 61-614 Poznań, Poland,

^2^ Department of Ecology, Swedish University of Agricultural Sciences, Box 7044, Se 750 07 Uppsala, Sweden

^3^ Department of Avian Biology and Ecology, Faculty of Biology, Adam Mickiewicz University, Umultowska 89, 61-614 Poznań, Poland

^4^ Institute of Nature Conservation, Polish Academy of Sciences, Mickiewicza 33, 31-120 Krakow, Poland,

^5^ Department of Invertebrate Zoology, Faculty of Biology and Environmental Protection, Nicolaus Copernicus University, Lwowska 1, 87-100 Toruń, Poland,

^6^ Department of Behavioural Ecology, Faculty of Biology, Adam Mickiewicz University, Umultowska 89, 61-614 Poznań, Poland

^7^ Institute of Plant Protection - National Research Institute Research Centre of Quarantine, Invasive and Genetically Modified Organisms, Wl. Wegorka 20 60-318 Poznan, Poland,

^8^ Institute of Zoology, Poznań University of Life Sciences, Wojska Polskiego 71C, 60-625 Poznań, Poland.

Author for correspondence: Zuzanna M. Rosin, E-mail: zuzanna.rosin@gmail.com, telephone number: +46 018 673347; Orcid ID: orcid.org/0000-0002-7565-7547

Table S1. Mean (± SE) frequencies of behaviour types of *C. nemoralis* per minute per 10 individuals studied in laboratory conditions. Abbreviations of behaviour types: see Table 1.

| Behaviour type | Morph | | | |
| --- | --- | --- | --- | --- |
|  | Pink  unbanded | Pink  mid-banded | Yellow unbanded | Yellow  5-banded |
| Inactive | 9.03±1.32 | 8.88±1.91 | 9.11±1.51 | 9.36±1.09 |
| Locomotion | 0.53±0.76 | 0.45±0.82 | 0.45±0.72 | 0.40±0.78 |
| Ground | 1.19±1.16 | 1.34±1.28 | 1.28±1.37 | 1.10±1.27 |
| Climbing | 5.76±1.63 | 4.40±2.08 | 5.71±1.95 | 4.98±2.04 |
| Shelter | 2.87±1.65 | 3.99±1.74 | 2.84±1.88 | 3.83±1.53 |
| Feeding | 0.12±0.24 | 0.10±0.32 | 0.10±0.26 | 0.05±0.18 |

Table S2. Mean (± SD), maximal and minimal values of variables describing micro-habitat and habitat of *C. nemoralis* natural colonies. Summary statistics are shown separately for micro-habitats with shells damaged by birds and mice present and absent. Variable abbreviations: % dead plants - % cover of dead plants in micro-habitat area (50 × 50 cm squares), % live plants - % cover of live vegetation in micro-habitat area, % ground - % cover of bare ground in micro-habitat area, Mean height of plants [cm]: in micro-habitat area(square), Distance to shrub [m] – distance to the nearest shrub from a study square, Distance to tree [m] – distance to the nearest tree from a square, % shrubs - % cover of shrubs in colony area (shading), thrush pair density – density of *Turdus* sp. pairs within 50 m radius from the colony centre, Rodent holes – number of rodent holes in colony area, Shells damaged by birds – number of shells damaged by birds found in colony area, Shells damaged by mice - number of shells damaged by birds found in colony area.

| Variable | Shells damaged by birds | | | | | | | | Shells damaged by mice | | | | | | | |
| --- | --- | --- | --- | --- | --- | --- | --- | --- | --- | --- | --- | --- | --- | --- | --- | --- |
|  | Absent | | | | Present | | | | Absent | | | | Present | | | |
|  | Mean | SD | Min | Max | Mean | SD | Min | Max | Mean | SD | Min | Max | Mean | SD | Min | Max |
| *Micro-habitat* |  |  |  |  |  |  |  |  |  |  |  |  |  |  |  |  |
| % dead plants | 35.67 | 24.23 | 0 | 100 | 28.45 | 20.56 | 0 | 98 | 35.87 | 24.13 | 0 | 100 | 32.45 | 25.10 | 0 | 50 |
| % live plants | 54.41 | 24.23 | 0 | 100 | 40.90 | 23.48 | 0 | 80 | 54.06 | 24.35 | 0 | 100 | 54.6 | 22.93 | 0 | 90 |
| % ground | 9.36 | 15.61 | 0 | 95 | 30.65 | 20.49 | 0 | 70 | 9.59 | 16.07 | 0 | 95 | 11.93 | 15.41 | 0 | 80 |
| Rodent holes | 0.15 | 0.50 | 0 | 6 | 0.06 | 0.23 | 0 | 1 | 0.15 | 0.51 | 0 | 6 | 0.13 | 0.40 | 0 | 2 |
| Mean height of plants | 8.22 | 0.63 | 6 | 39.5 | 8.2 | 0 | 8.2 | 8.2 | 8.22 | 0.65 | 6 | 39.5 | 8.22 | 0.31 | 8.2 | 13.8 |
| Distance to shrub | 13.75 | 14.73 | 0 | 45 | 4.66 | 9.41 | 0 | 40 | 13.57 | 14.70 | 0 | 45 | 13.51 | 14.71 | 0 | 40 |
| Distance to tree | 15.91 | 14.40 | 0 | 65 | 11.36 | 17.41 | 0 | 45 | 15.53 | 14.34 | 0 | 60 | 18.4 | 15.50 | 0 | 65 |
|  |  |  |  |  |  |  |  |  |  |  |  |  |  |  |  |  |
|  | All colonies | | | |  |  |  |  |  |  |  |  |  |  |  |  |
| *Habitat (colony)* | Mean | SD | Min | Max |  |  |  |  |  |  |  |  |  |  |  |  |
| % shrubs | 15.24 | 21.43 | 0 | 90 |  |  |  |  |  |  |  |  |  |  |  |  |
| Shells damaged by mice | 1.84 | 3.81 | 0 | 23 |  |  |  |  |  |  |  |  |  |  |  |  |
| Shells damaged by birds | 1.12 | 4.37 | 0 | 36 |  |  |  |  |  |  |  |  |  |  |  |  |
| Thrush pair density | 0.44 | 0.80 | 0 | 3 |  |  |  |  |  |  |  |  |  |  |  |  |
| Temperature | 18.61 | 5.61 | 7.0 | 32.8 |  |  |  |  |  |  |  |  |  |  |  |  |
| Humidity | 63.90 | 13.75 | 33.0 | 99.0 |  |  |  |  |  |  |  |  |  |  |  |  |

Table S3. Spearman correlations between continuous variables describing habitat and micro-habitat of *C. nemoralis* natural colonies. Variables’ names explanation: see Table S2.

| Variable | (1) | 2 | 3 | 4 | 5 | 6 | 7 | 8 | 9 | 10 | 11 |
| --- | --- | --- | --- | --- | --- | --- | --- | --- | --- | --- | --- |
| (1) % dead plants | 1 | -0.75 | -0.34 | -0.11 | 0.07 | 0.11 | 0.00 | -0.17 | 0.14 | -0.09 | 0.03 |
| (2) % live plants |  | 1 | -0.31 | 0.09 | 0.12 | 0.00 | 0.04 | 0.12 | -0.20 | 0.03 | -0.13 |
| (3) % ground |  |  | 1 | 0.04 | -0.26 | -0.14 | -0.03 | 0.08 | 0.05 | 0.13 | 0.15 |
| (4) Rodent holes |  |  |  | 1 | -0.03 | 0.03 | 0.09 | 0.06 | -0.13 | -0.02 | -0.02 |
| (5) Mean height of plants |  |  |  |  | 1 | 0.18 | -0.06 | -0.08 | 0.03 | -0.02 | -0.02 |
| (6) Distance to shrub |  |  |  |  |  | 1 | 0.51 | -0.20 | 0.01 | -0.11 | -0.31 |
| (7) Distance to tree |  |  |  |  |  |  | 1 | -0.15 | 0.01 | -0.12 | -0.49 |
| (8) Temperature |  |  |  |  |  |  |  | 1 | -0.27 | 0.10 | 0.15 |
| (9) Humidity |  |  |  |  |  |  |  |  | 1 | -0.02 | 0.01 |
| (10) Thrush pair density |  |  |  |  |  |  |  |  |  | 1 | 0.09 |
| (11) % shrubs |  |  |  |  |  |  |  |  |  |  | 1 |

Table S4. Number of individuals of *C. nemoralis* morphs (N), % of individuals that climbed up and hid in shelter, observed in 50 × 50 cm squares, where shells damaged by birds and mice were present or not (n – number of examined squares).

| Morph | Shells damaged by birds | | | | | | Shells damaged by mice | | | | | |
| --- | --- | --- | --- | --- | --- | --- | --- | --- | --- | --- | --- | --- |
|  | Absent  n = 622 squares | | | Present  n = 23 squares | | | Absent  n = 553 squares | | | Present  n = 92 squares | | |
|  | N | Climb  [%] | Shelter  [%] | N | Climb  [%] | Shelter  [%] | N | Climb  [%] | Shelter  [%] | N | Climb  [%] | Shelter  [%] |
| Brown unbanded | 164 | 11.5 | 20.7 | 1 | 0 | 100 | 141 | 12.8 | 22.7 | 24 | 4.2 | 12.5 |
| Brown mid-banded | 21 | 14.3 | 0 | 0 | 0 | 0 | 19 | 10.5 | 0 | 2 | 50.0 | 0 |
| Pink unbanded | 298 | 11.1 | 17.5 | 5 | 0 | 20.0 | 262 | 11.5 | 17.6 | 41 | 7.3 | 17.1 |
| Pink mid-banded | 489 | 12.5 | 14.5 | 8 | 25.0 | 87.5 | 464 | 12.7 | 15.5 | 33 | 12.1 | 18.2 |
| Pink 3-banded | 113 | 7.1 | 15.9 | 4 | 25.0 | 50.0 | 103 | 7.8 | 15.5 | 14 | 7.1 | 28.6 |
| Pink 5-banded | 173 | 4.6 | 9.8 | 3 | 33.3 | 66.6 | 164 | 4.9 | 9.7 | 12 | 8.3 | 25.0 |
| Yellow unbanded | 346 | 15.2 | 22.8 | 10 | 30.0 | 70.0 | 309 | 16.2 | 26.2 | 46 | 10.9 | 10.9 |
| Yellow mid-banded | 725 | 9.1 | 12.1 | 11 | 9.1 | 63.6 | 669 | 8.8 | 13.0 | 67 | 11.9 | 11.9 |
| Yellow 3-banded | 139 | 5.6 | 16.5 | 5 | 0 | 0 | 131 | 6.1 | 16.8 | 13 | 0 | 7.7 |
| Yellow 5-banded | 717 | 8.8 | 16.7 | 22 | 9.1 | 72.7 | 669 | 8.5 | 18.2 | 70 | 11.4 | 20.0 |
